# Correction

**DOI:** 10.1111/cas.15675

**Published:** 2022-12-09

In an article^1^ titled “Interleukin 6 regulates the expression of programmed cell death ligand 1 in thyroid cancer” by Guo‐Qiang Zhang, Qiong Jiao, Chen‐Tian Shen, Hong‐Jun Song, Hui‐Zhen Zhang, Zhong‐Ling Qiu, Quan‐Yong Luo, there were errors in Figure 6 and Figure 5 legend.

The revised Figure 5 legend is as follow:

FIGURE 5 Interleukin 6 (IL‐6) upregulated programmed cell death ligand 1 (PD‐L1) expression through the mitogen‐activated protein kinase (MAPK) pathway. A, In TPC‐1 and BAPCP, the total protein levels of extracellular signal–regulated kinase (ERK), C‐Jun NH(2)–terminal kinase (JNK), signal transducers and activators of transcription 3 (STAT3) and serine/threonine‐protein kinase (AKT) did not change in response to IL‐6. However, the phosphorylation levels of ERK, JNK, STAT3 were significantly increased, while the phosphorylation level of AKT was not. Considering that the cell lines and treatments corresponding to each lane were identical, the same reference protein band was accepted in both panels to better visually compare changes in the target proteins relative to the reference protein (total target protein/reference protein, phosphorylated target protein/reference protein, and phosphorylated target protein/total target protein). B, After treatment with U0126 (ERK inhibitor) or SP600125 (JNK inhibitor), the IL‐6–induced total PD‐L1 protein reduced, and combination of them had a synergistic effect on PD‐L1 expression. C, Flow cytometry result showed that the membrane PD‐L1 expression increased after IL‐6 treatment but decreased after subsequent treatment with U0126 or SP600125. D, The level of PD‐L1 mRNA in the IL‐6 group was higher than that in the control group. It was inhibited after treatment with U0126 or SP600125 which worked synergistically. # Indicates *p* < 0.05. *Indicates *p* ≥ 0.05.

The revised Figure 6A is shown below:

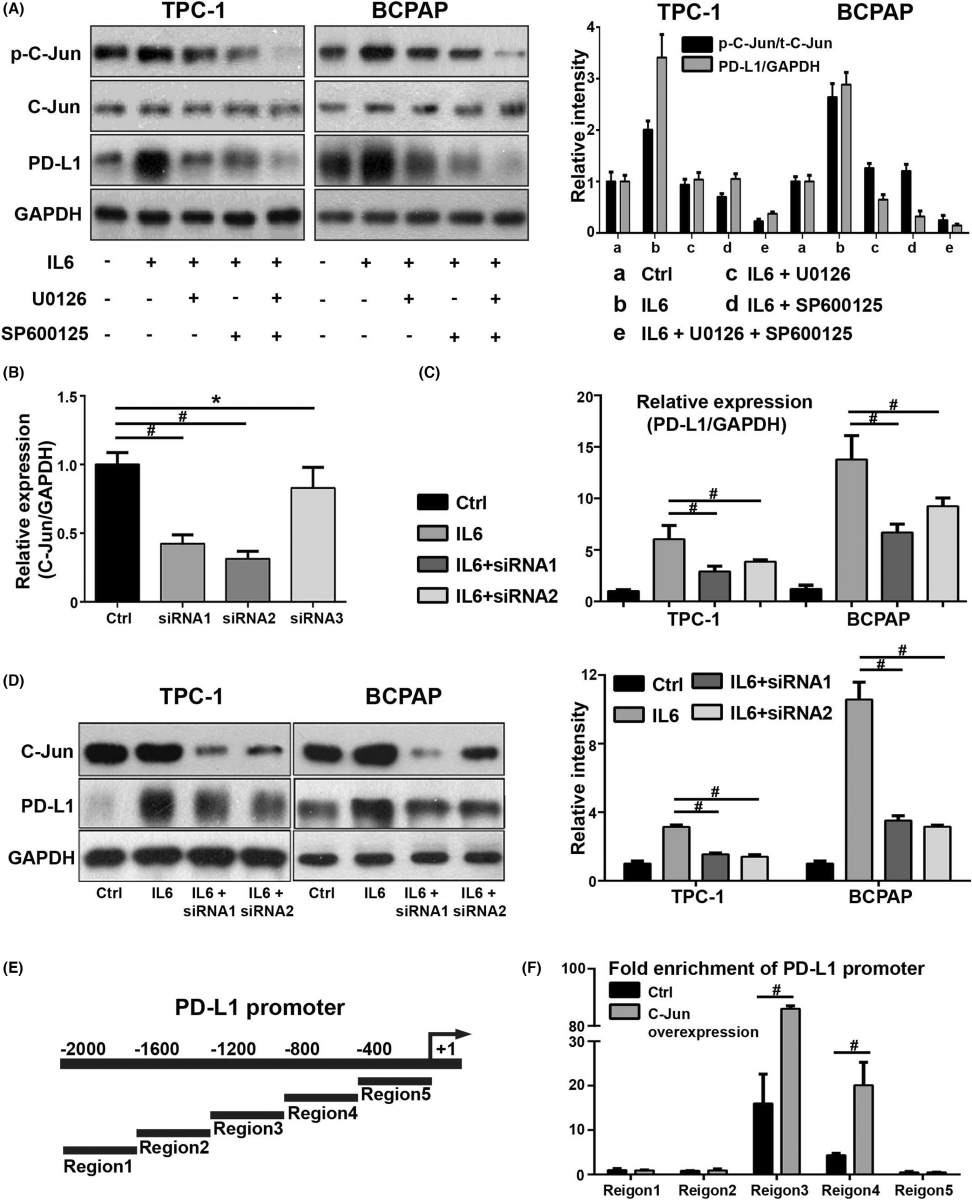

The authors apologize for the error.
